# Deep Learning in Population Genetics

**DOI:** 10.1093/gbe/evad008

**Published:** 2023-01-23

**Authors:** Kevin Korfmann, Oscar E Gaggiotti, Matteo Fumagalli

**Affiliations:** Professorship for Population Genetics, Department of Life Science Systems, Technical University of Munich, Germany; Centre for Biological Diversity, Sir Harold Mitchell Building, University of St Andrews, Fife KY16 9TF, UK; Department of Biological and Behavioural Sciences, Queen Mary University of London, UK

**Keywords:** population genetics, machine learning, artificial neural networks, simulations, balancing selection

## Abstract

Population genetics is transitioning into a data-driven discipline thanks to the availability of large-scale genomic data and the need to study increasingly complex evolutionary scenarios. With likelihood and Bayesian approaches becoming either intractable or computationally unfeasible, machine learning, and in particular deep learning, algorithms are emerging as popular techniques for population genetic inferences. These approaches rely on algorithms that learn non-linear relationships between the input data and the model parameters being estimated through representation learning from training data sets. Deep learning algorithms currently employed in the field comprise discriminative and generative models with fully connected, convolutional, or recurrent layers. Additionally, a wide range of powerful simulators to generate training data under complex scenarios are now available. The application of deep learning to empirical data sets mostly replicates previous findings of demography reconstruction and signals of natural selection in model organisms. To showcase the feasibility of deep learning to tackle new challenges, we designed a branched architecture to detect signals of recent balancing selection from temporal haplotypic data, which exhibited good predictive performance on simulated data. Investigations on the interpretability of neural networks, their robustness to uncertain training data, and creative representation of population genetic data, will provide further opportunities for technological advancements in the field.

**Significance**

Deep learning, a powerful class of supervised machine learning, is emerging as a promising inferential framework in evolutionary genomics. In this review, we introduce all deep learning algorithms currently used in population genetic studies, highlighting their strengths, limitations, and empirical applications. We provide perspectives on their interpretability and usage in face of data uncertainty, whilst suggesting new directions and guidelines for making the field accessible and inclusive.

## From Model-Based to Data-Driven Discipline

Population genetics arose in the early 20th century as a conceptual framework aimed at unifying two opposing views of evolution (Provine 2020). As such, it developed a rich body of theory that became a vast treasure trove of probabilistic models to develop sophisticated statistical methods when molecular data became available. This body of theory has continued to grow in complexity in order to consider more realistic evolutionary and genetic scenarios as well as more efficient computational algorithms. Therefore, the field of population genetics has been dominated by model-based statistical approaches. One could even say that many population geneticists would agree to the proposition of slightly modifying George E.P. Box’s aphorism so as to say that in our field, all models are wrong but ***many*** are useful.

The preeminence of model-based statistical inference may explain the fact that our field has lagged behind other life-science disciplines in the adoption of machine learning methods and, in particular, deep learning approaches. Clearly, the black-box nature of deep learning is an important obstacle to applications in the domain of population genetics, which main objective is to uncover the genetic and evolutionary mechanisms responsible for the diversity of life on our planet. Another deterrent is the apparent difference in foci between the fields of statistics and machine learning. Statistics is focused on inference through the creation and fitting of a probabilistic model while machine learning is focused on prediction using general-purpose algorithms that capture patterns present in complex and large data sets (Bzdok et al. 2018). However, population geneticists are interested in both inference and prediction, as clearly illustrated by the general interest in making inferences about demographic history of species on the one hand and detecting signatures of natural selection or assigning individuals to populations on the other. Nevertheless, most genetic clustering methods and so-called genome scans of selection are based on probabilistic models, in some cases mechanistic [e.g. Bayescan (Foll and Gaggiotti 2008) and STRUCTURE (Pritchard et al. 2000)] and in others phenomenological [e.g. LFMM (Frichot et al. 2013) and DAPC (Jombart et al. 2010)].

The focus on model-based statistical inference in population genetics has been challenged by the massive data sets generated by next-generation sequencing technologies (Levy and Myers 2016). This is particularly the case for maximum-likelihood and Bayesian methods, which are implemented using expensive computational methods such as Monte Carlo Markov Chain and Expectation-Maximization. In principle, the computational cost of calculating the likelihood function of very complex models, can be overcome using Approximate Bayesian Computation (ABC), which relies on the use of summary statistics to capture the information present in raw population genetic data (Bertorelle et al. 2010). In ABC, the posterior distribution of the parameter(s) to be estimated is approximated without the calculation of a likelihood function. Instead, a model fit is obtained by the collection of simulated summary statistics matching the observed values (Beaumont et al. 2002). ABC has been widely and successfully used for population genetic inferences (Lopes and Beaumont 2010). However, capturing enough information requires large numbers of summary statistics which lead to a “curse of dimensionality” because, as the number of summary statistics increases, the error in the approximation increases (Prangle 2015). This problem has led to an increasing interest in machine learning approaches (Schrider and Kern 2018). The underlying rationale here is that analysing genomic data with machine learning methods can uncover signatures of evolutionary and genetic processes in a model agnostic way and in doing so teach us something new about nature (Schrider and Kern 2018). But a major motivation for the shift is the practical reality that population genetics has been transitioning from a theory-driven discipline into a data-driven field with vast amounts of genomes and metadata at hand in the past few years. For instance, in human population genetics, scientists have access to high-quality whole-genome sequencing data from more than 150,000 individuals from the UK Biobank (Halldorsson et al. 2022), and more than 3,000 individuals distributed world-wide (Byrska-Bishop et al. 2022), or to hundreds of genomic data from ancient samples (https://reich.hms.harvard.edu/datasets).

In this review, we will focus on a particular subset of supervised machine learning algorithms, namely deep neural networks. Although such methods can be considered as the epitome of a black box, we will argue that new advances in this field are providing the tools we need to uncover the mechanisms underlying the complex patterns present in population genomic data. Moreover, deep learning can be implemented to analyse raw genetic data as well as summary statistics. Additionally, it has been used to carry out statistical inference about the demographic history of populations as well as to carry out selection scans and assign individuals to geographic locations. Applications to demographic history inference embrace the model-based tradition of population genetics in that the training set (see Glossary) is usually generated through simulations of specific evolutionary scenarios. Applications to genome scan methods on the other hand, rely on new techniques for evaluating the importance of features, in this case loci, in predicting an outcome such as a phenotype or an environmental factor that may exert a selective pressure.

We will first provide a definition of supervised machine learning and its applications in population genetics. We will then focus our attention on various deep learning algorithms currently used in the field, with a discussion on efforts to “open the black box” of said algorithms. We will finally discuss ongoing challenges of deep learning applications in population genetics, and highlight future research directions.

## Machine Learning in Population Genetics

Machine learning, a subset of artificial intelligence, refers to a class of operations using data to perform inferential tasks without explicit mathematical models. To do so, machine learning algorithms identify informative patterns which can be then used to predict unknown outcomes. Typically, the performance of machine learning algorithms increases with the amount of available data. Machine learning comprises both supervised and unsupervised algorithms. Unsupervised machine learning aims at finding patterns and clusters within the data, and does not have a notion of prediction. On the other hand, supervised machine learning algorithms automatically tune their internal parameters to maximize the prediction accuracy and, as such, require a known data set (called training set) to learn the relationship between input and output.

To train a supervised machine learning algorithm, the available data sets are typically divided into training, validation, and testing sets, with the latter two sets used to evaluate the performance during and after training. In supervised learning, a labeled data set (which explicitly relates any given input to a specific output) is given to the algorithm. The loss (the distance between the predicted and true value) is calculated, and at the next iteration the internal parameters are updated towards decreasing loss (and increasing accuracy). Training a supervised machine learning algorithm is a fine balance between prediction accuracy over the training set and generalization performance over the testing set.

Machine learning has a rich history in biological sciences and genomics (reviewed in Yue and Wang 2018; Zou et al. 2019; Greener et al. 2022). Additionally, supervised machine learning methods have been designed and deployed to perform population genetic tasks such as variant calling (Poplin et al. 2018) and the prediction, characterization, and localization of signatures of natural selection (Pavlidis et al. 2010; Lin et al. 2011; Ronen et al. 2013; Pybus et al. 2015; Schrider and Kern 2016; Sugden et al. 2018; Mughal and DeGiorgio 2019; Koropoulis et al. 2020). An important difference between the variant calling application (which only uses observed data) and those aimed at detecting selection is that the latter implement an innovation first introduced by Pavlidis et al. (2010) whereby the ML algorithms are trained using synthetic data sets generated via simulations. These applications, therefore, can be considered as being part of likelihood-free simulation-based approaches (Cranmer et al. 2020), which are commonly employed in population genetics. Currently, most population genetics applications of ML use this strategy but, as we describe below, some recent applications only use observed data to train the algorithms. These applications, however, require the combination of genotypic data with phenotypic, environmental or geographic coordinate data.

As already stated, in this review we will focus on deep learning, a class of machine learning algorithms based on artificial neural networks comprising nodes in multiple layers connecting features (input) and responses (output) (LeCun et al. 2015). Weights between nodes are optimized during the training to minimize the distances between predictions and ground truth. After training, an ANN can predict the response given any arbitrary new input data. Unlike approaches that use a predefined set of summary statistics as input, deep learning algorithms can effectively learn which features are sufficient for the prediction (LeCun et al. 2015). This is an important aspect as summary statistics are meaningful but human-constructed features. When dealing with different sources of raw data, the design of such features has been a major part of information engineering. A key finding of deep learning was that such features emerged within a well-trained deep network: they are effectively suggested and discovered by a network during training (Krizhevsky et al. 2012). This finding has been repeated in different domains: features can be automatically discovered, and new suggestions made, by the approaches of deep learning. Nodes in an ANN can be arranged in various numbers and layers, making this method as flexible and “deep” as needed.

Deep learning in population genetics is in its infancy, and most of current applications rely on synthetic data sets for training. Nevertheless deep learning represents a notable progress over commonly used simulation-based techniques for several reasons. First, they have the capacity to handle any feature extracted from a data set as input and are less sensitive to poorly crafted summary statistics than ABC (Csilléry et al. 2010). Second, neural networks are universal approximators of any complex function provided that they include a sufficiently large number of “neurons,” non-linear units (Hornik et al. 1989). Nevertheless, careful monitoring of networks’ training and a posteriori diagnostic analyses are required to ensure that predictions are robust.

Whilst overviews of machine learning applications for population and molecular genetics are provided elsewhere (Schrider and Kern 2018; Fountain-Jones et al. 2021; Kumar et al. 2022), here we aim at providing an update on the latest advances in deep learning algorithms and how they have been exploited to address questions in population genetics. Additionally, we focus our attention on deep neural networks, in all their supervised forms, rather than including other commonly used algorithms such as support vector machine (Pavlidis et al. 2010), random forests (Schrider and Kern 2016; Vizzari et al. 2020), gradient forests (Laruson et al. 2022), and hierarchical boosting (Pybus et al. 2015). Finally, we restrict our review on applications in population genomics while acknowledging that similar algorithms herein described are used in other related disciplines like genomics (Yue and Wang 2018), phylogenetics (Suvorov et al. 2020; Azouri et al. 2021; Blischak et al. 2021), phylogeography (Fonseca et al. 2021; Perez et al. 2022), and epidemiology (Voznica et al. 2021).

**Glossary**

- **Accuracy:** proportion of correct predictions made by a model
- **Activation function:** operation that each neuron performs
- **Attribute:** name of a variable describing an observation
- **Bias term:** a term attached to neurons allowing the model to represent patterns that do not pass through the origin
- **Backpropagation:** gradient descent-based learning algorithm for calculating derivatives through the network starting from the last layer
- **Confusion Matrix:** table that summarizes the prediction performance by providing false and true positive/negative rates
- **Embedding:** learned low-dimensional continuous vector representation of a concept (e.g. a word, sentence, genotype matrix or graph)
- **Epoch:** the number of times the algorithm sees the data set
- **Feature:** input variable used in making predictions
- **Hyperparameters:** higher level properties of a model controlling the training process (e.g. learning rate, number of epochs) and that need to be tuned, in principle before the ML model is trained
- **Instance:** a data point or sample in a data set (observation)
- **Learning rate:** magnitude at which an algorithm updates its parameters
- **Loss:** (also called cost) measurement of distance between predictions and ground truth; its function is minimized during training
- **Normalization:** scaling technique used when input features have different ranges
- **Regularization:** an additional penalty to the loss function for better generalization
- **Testing set:** portion of the data set that it is not used for training, but rather to evaluate the performance a neural network
- **Training set:** portion of the data set that it is used to optimize parameters of a neural network
- **Tuning or hyperparameter optimization:** process of finding the hyperparameter values that maximize the performance of the model
- **Validation set:** portion of the data set that it is used for monitoring the training of a neural network

## Deep Learning Algorithms

We now introduce, describe and discuss four common families of architectures for deep learning algorithms used in population genetics: fully connected neural networks, convolutional neural networks, recurrent neural networks, and generative models. For each type of algorithm, we illustrate their main applications in the field and the novel findings generated by their deployments. Note that these general algorithms have a long history spanning many decades and numerous original contributions which we cannot properly credit in our review because of space. Thus, we refer readers interested in historical developments to previous publications (Schmidhuber 2014).

### Fully Connected Neural Networks

**Fig. 1.**
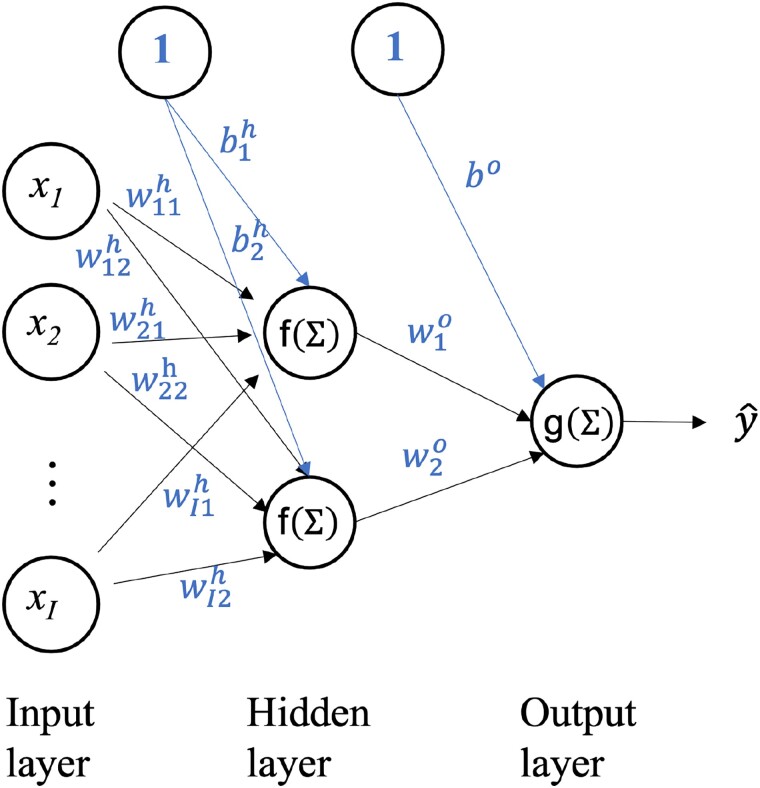
A simple FCNN consisting of a single hidden layer with only two nodes. f and g represent different activation functions used respectively in the hidden layer and the output layer and h and o superscripts are used to identify parameters associated with these layers; all other parameters are defined in the text.

Fully connected neural networks (FCNNs) are suitable for generic prediction problems when there are no special relations among the input data features. They can be viewed as a generalization of linear regression. In fact, standard regression is nested in the general neural network framework in the sense that a linear regression fits a hyperplane to the data, while a neural network fits a space of hyperplanes in a transformed space (Qin et al. 2022). This becomes clear by comparing the formulation for the simplest multivariate linear regression model with the equation representing the operations taking place in a single node of a hidden layer of an FCNN,

linear regression: $y_{i}(x,w)=b+\sum_{i=1}^{I}w_{i}x_{i}$
FCNN: $s(x,w)=f(b+\sum_{i=1}^{I}w_{i}x_{i})$,

where b is the bias (not to be confounded with statistical bias), $w=\{w_{i}\}$ is a vector of weights, $x=\{x_{i}\}$ is a vector of input features (explanatory variables), and f is a nonlinear activation function. In an FCNN with a single hidden layer, there will be a number J of hidden nodes, each carrying out a similar operation using a different vector of weights, all of which can be represented by a matrix $W=\{w_{ij}\}$, i=1,2…,I, j=1,2,…J. A very simple example of an FCNN with one hidden layer and only two nodes is presented in figure 1.

In the linear regression case a dependent variable is computed by calculating the dot-product of a set of input data points with a set of parameters. This output variable is then used in the context of a maximum-likelihood or least-square approach to optimize the set of learnable parameters. FCNNs extend this idea by computing a matrix-product of the weight matrix with the input data points, which is then transformed with a non-linear activation function. The activation function is applied element-wise and the result is called an embedding. Instead of using the maximum-likelihood or least-square approaches for optimization, FCNNs are optimized using the multivariate version of the gradient-descent algorithm, which iteratively adapts the parameters across the network layers [back-propagation algorithm (Linnainmaa 1976; LeCun et al. 1989)] based on a task-specific loss-function and learning rate. A fundamental property of FCNNs is expressed by the Universal Approximation Theorem, which states that a neural network with a single hidden-layer can approximate any continuous function to any desired precision. Precision can be increased by increasing the number of hidden neurons or the number of hidden layers. It is this property that enables the use of neural networks as a viable alternative to common model-based statistical methods.

In an early application of deep learning methods to population genetics, FCNNs are used to simultaneously infer natural selection and population bottlenecks (Sheehan and Song 2016). This approach was inspired by ABC methods and therefore used summary statistics to extract the information present in the raw data, which was then fed to a fixed-size linear input layer of the network. To discriminate between demographic and natural selection effects, Sheehan and Song trained the FCNN using simulated data sets generated under various models assuming different bottleneck times and selection models (Sheehan and Song 2016). The software evoNet, which implemented said FCNN, was applied to almost 200 genomes of *Drosophila melanogaster* from Africa to jointly infer the demography history and loci under selection. One interesting analysis in the study is the evaluation of the most informative summary statistics, either by permutation or perturbation. Notably, summary statistics derived from the site frequency spectrum, linkage disequilibrium (LD), number and location of single-nucleotide polymorphisms (SNPs), and identity-by-state tracts are among the most important features for the inference of population size changes and type of selection.

Another example of an FCNN application in population genetics that uses simulated data to train the algorithm is provided by the work of Burger and colleagues on the estimation on mutation rates (Burger et al. 2022). They show that a simple neural network is able to recapitulate estimators of mutation rate for intermediate recombination rates. As a novel methodological advance, their implementation features an adaptive reweighting of the loss function based on model-based estimators of the mutation rate. By doing so, with sufficient and appropriate training set, only a single hidden layer is required to achieve the same performance of model-based estimators. The method was able to recover variation in mutation rates from synthetic human population genetic data under a realistic recombination map.

There are also recent population genetics applications of FCNNs that implement the standard approach of training algorithms using observed instead of simulated data. A good example is Locater, which assigns individual genotypes to their geographic origin (Battey et al. 2020). Interestingly, this method implements a regression approach that is capable of assigning correlated genetic samples to similar geographic space. Uncertainty in the estimates due to drift is taken into account by running predictions in windows across the genome. Simulations indicate that Locater has an accuracy comparable to that of other state-of-the-art competing algorithms but with shorter run-times. Its application to an empirical population genetic data set of *Anopheles* mosquitoes, *Plasmodium falciparum*, and human populations, provides results that are in general concordant with current knowledge.

Another example that only uses observed data to train the FCNN is DeepGenomeScan (Qin et al. 2022). However, this method’s objective departs from the prevalent use of neural networks, that is prediction and pattern recognition. Its aim is to develop a statistical framework to carry out genome scans or GWAS, much in the same way that PCA and redundancy analysis have been used to develop equivalent approaches (Luu et al. 2017; Capblancq et al. 2018). Specifically, DeepGenomeScan implements an FCNNs that uses genotypes to predict individuals’ traits (e.g. geographic coordinates or phenotype), and constructs a feature importance measure based on the weights of the trained network. Furthermore, *P*-values for variable importance are obtained through bootstrapping of the input. As opposed to other methods that can only detect linear associations, DeepGenomeScan is able to detect non-linear ones thanks to the non-linear approximation property of FCNNs. Its application to a genomic data set of human samples of European ancestry identified novel targets of natural selection which showed significant geographic variation.

Finally, we note that FCNNs have also been used in the context of ABC frameworks. Early studies used neural networks to construct the posterior distribution of parameters from the collection of accepted values (Blum and François 2010), as implemented in the abc package (Csilléry et al. 2012). More recently, Mondal and colleagues coupled an ABC framework, using the site frequency spectrum (SFS) as summary statistic, with a four-layer FCNN to infer the demographic history of human Eurasian populations (Mondal et al. 2019). Their implementation includes an *ad hoc* noise injection algorithm to partly take into the account any bias associated with a simulated training set. A similar study by Villanea and Schraiber used the joint SFS between Europeans and Neanderthal genomes to fit a demographic model using a 3-layer FCNN (Villanea and Schraiber 2019). Both studies inferred multiple gene flow events between archaic and anatomically modern humans.

Summary statistics and genotype matrices are not the only way in which population genomic data can be described and used as input to deep learning algorithms. It is also possible to represent samples of sequences as images and, in the next section, we discuss an architecture that is being increasingly applied to such data.

### Convolutional Neural Networks

**Fig. 2.**
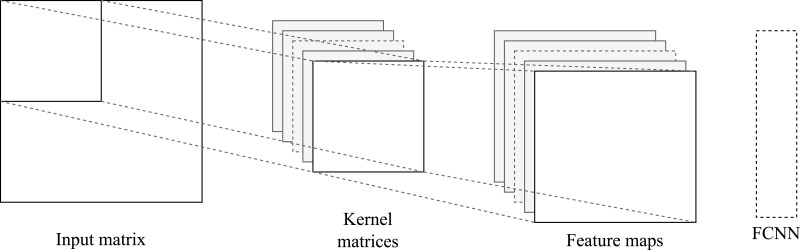
A simple CNN illustration consisting of the input matrix (i.e. genotype matrix), a user-specified number of kernels (or filters) and the resulting feature maps, followed by an FCNN.

Convolutional neural networks (CNNs) are specifically designed to analyse data that has a grid-like structure, such as images (LeCun et al. 2004; Krizhevsky et al. 2012). Whilst in theory FCNNs could be used to make predictions from images, the number of features (i.e. pixels) they contain would require networks with a very large number of parameters, which would render them very slow and computationally expensive. Similarly to FCNNs, CNNs are comprised of a set of learnable parameters (LeCun et al. 1989; LeCun and Bengio 1995). However, as opposed to FCNNs, in which hidden layers are all of the same type (layers of neurons carrying out similar operations), CNNs architecture consists of consecutive sets of convolutional and pooling layers, followed by a fully connected set of layers (similar to an FCNN, fig. 2). The first convolutional layer takes the input image and carries out a convolution using a kernel (also known as filter; a matrix of learnable parameters) to generate a feature map that is then fed to the pooling layer. This layer uses a filter to reduce the size of the feature map and to help dissociate a particular feature from its position in the input image. This first set of operations will capture coarse grained features; adding additional convolutional and pooling layers helps capture more fine-grained features (O’Shea and Nash 2015). The final step of the convolutional layers (flatten step) converts the feature map into a vector that is fed to the fully connected layers that will carry out the image classification step. The number of kernels, their dimensions, and initialization are all hyperparameters of the model.

CNNs can be regarded as a regularized version of FCNNs with a focus on localized spatial signatures. In fact, a fundamental property of CNNs is the space-invariance of the learned features in the data set, which means that they can identify a pattern regardless of its spatial location in the image. Note, however, that identification of feature realizations like rotations or scaling requires either appropriate samples or perturbations of the input (Goodfellow et al. 2016).

First applications of CNNs in population genetics relied on “image” data sets in the form of stacked summary statistics. The method implemented in software diploS/HIC aimed at classifying genomic windows into neutral regions or under soft or hard selective sweeps from unphased genotypes (Kern and Schrider 2018). It did so by applying convolutional operations on a feature vector of normalized summary statistics calculated in windows surrounding the target location. The architecture consisted of three branches of two-dimensional convolutional layers with different filter sizes, followed by max pooling, flattening and two fully connected layers. Extensive simulations of tested scenarios were produced to train the CNN. The authors showed that CNNs outperformed competing ML algorithms previously used for this classification task (Schrider and Kern 2016), possibly because CNNs retain the spatial relationships of summary statistics. Notably, with moderate sample size, diploS/HIC appears to be robust to model misspecification as it retains accuracy when predictions for a population growth demography were obtained from CNNs trained on constant size population simulations. As an application of diploS/HIC, the authors replicated previous findings of selective sweep in the *Anopheles gambiae* genome. A later extension of this method led to partialS/HIC which uses CNNs on a larger feature vector of summary statistics for a finer classification of selective events, including partial sweeps and linked selection (Xue et al. 2020). Finally, an additional application of CNNs based on summary statistics to test against different modes of selective sweeps has been recently proposed (Caldas et al. 2022). This study uses varying window sizes to accommodate the calculation of summary statistics at different genomic extents within the target loci. They also introduced a hybrid simulation strategy to pair the flexibility of forward-in-time simulations with the efficiency of coalescent ones.

An approach that fully exploits the potential of CNNs is to replace summary statistics as input with full information on sequence alignments, with convolutional layers automatically extracting informative features. Input data can consist of either genotype or haplotype sequences. In the simplest form, input data are a binary matrix, with rows and columns corresponding to individuals and alleles at each SNP, respectively. Under this representation, and in opposition to the structured nature of “classic” images, the ordering of individuals (i.e. random samples from a population) in an unstructured population is arbitrary and carries no information (Chan et al. 2018); i.e. genetic data are exchangeable. However, standard CNNs rely on spatial information and, therefore, the ordering of the data can affect its accuracy. To avoid this problem, individuals need to be sorted in a “biologically meaningful” way. For example, Flagel and collaborators sort chromosomes by genetic similarity (Flagel et al. 2018). Additionally, they represent the information on genomic positions of SNPs as a separate branch in the architecture. Interestingly, the inclusion of monomorphic sites in windows of fixed length seems to yield good accuracy for predicting natural selection, as shown in a separate study (Nguembang Fadja et al. 2021). Notably, several applications of the proposed method are illustrated, with CNN achieving equal if not better performance than state-of-the-art methods to detect gene flow and selective sweeps, estimate recombination rates, and infer demographic parameters (Flagel et al. 2018). Therefore, these findings demonstrated the capability of CNNs to infer population genetic parameters, even in cases where a theoretical framework is not available.

To address the exchangeability issue, Chan et al. (2018) proposed an exchangeable neural network. This architecture consists of convolutional layers with 1-dimension kernels with a subsequent permutation-invariant function to allow for the network to be insensitive to the order of individuals. Although they employed the mean operation as permutation-invariant function, other functions are possible, including a fully connected layer. Another important contribution of this study is the adoption of a “simulation-on-the-fly” approach: training data is continuously generated by simulations to avoid the network to see the same data twice and therefore to reduce overfitting. This is a valuable consideration since, when reliable simulators are available (as in the case of population genetics), we have access to theoretically infinite training data, the latter being constrained by computing time only. The implemented software defiNETti was applied to illustrate the accuracy of exchangeable neural networks to predict recombination hotspots in human data.

Further solutions to tackle the issue of exchangeable genetic data have been explored by Torada et al. (2019) in the software ImaGene. Specifically, the authors showed how ordering haplotypes and SNPs by frequency leads to accurate predictions of positive selection. Whilst sorting SNPs implied a loss of information on LD patterns, this approach makes training faster with minimal decay in accuracy, as the number of learnable parameters is drastically reduced as the final fully connected layer is not required. However, double-sorting makes the method less appropriate for a general-purpose methodology. Additionally, by training and testing ImaGene with simulations conditioned on different demographic models, the authors quantified the drop in accuracy when CNNs are affected by model misspecification during training. Finally, a multiclass classification approach was proposed as an alternative method to approximate the posterior distribution of the selection coefficient, a continuous parameter typically hard to estimate.

In another landmark study, Sanchez et al. (2021) provide a comprehensive framework for building deep neural networks taking into account several nuances of the input data, such as the variable number of SNPs, their correlation, and the exchangeability of individuals. These challenges were tackled by proposing an architecture, called SPIDNA (Sequence Position Informed Deep Neural Architecture), which consisted of stacks of multiple blocks of convolutional, pooling, and fully connected layers. In addition to deploy their method to reconstruct changes in effective population size of cattle breed populations, the authors compared the accuracy of several deep neural networks against ABC, including hybrid approaches. Notably, results suggest that integrating deep learning with ABC marginally improves performance, and possibly explainability. Further investigations from the same authors demonstrated a more prominent increased performance using deep neural networks (Sanchez 2022). These studies depart from previous attempts to adapt existing architectures, and instead they suggest to build novel architectures tailored to the specifics of population genetic data.

In a later study, Gower et al. (2021) aimed to identify signatures of adaptive archaic introgression in the human genome without relying on statistics that capture the frequency of putatively introgressed haplotypes. The authors developed a deep learning method based on CNNs, genomatnn, to jointly infer archaic admixture and positive selection. genomatnn is trained from a matrix consisting of concatenated genotype alignments encompassing donor (archaic humans) and recipient (modern humans) populations. Matrix entries represent counts of minor alleles in an individual haplotype within a given genomic window. Thus, this approach is applicable to low-quality sequencing data where genotype calling can be bypassed by the statistical estimation of allele frequencies (Kim et al. 2011). Additionally, the authors proposed a framework to visually inspect the input features that are more informative for the prediction by means of saliency maps (Simonyan et al. 2013). Intriguingly, the latter indicated that the network focus most of its attention on Neanderthal and European haplotypes when exposed with data from an adaptive introgression, in line with the expected pairing of donor and recipient populations.

DeepSweep is another application of CNNs to detect selective sweeps from “haplotypic” images, as defined by the authors (Deelder et al. 2021). This method selects the longest common haplotype among neighboring SNPs, and sort all remaining haplotypes based on their distance to it. This sorted alignment of haplotype differences is then fed into a series of convolutional layers. The aim of the original study was to detect signatures of positive selection in malaria parasites, namely *Plasmodium falciparum* and *Plasmodium vivax*. Interestingly, the algorithm was then trained using real data from regions covering SNPs previously associated with drug resistance, and the validation was performed using a leave-one-out approach. Possibly as a result of both the data processing and training strategies, when deployed on whole-genome data, DeepSweep predicted selection targets to be known drug-resistance genes and largely overlapping with predictions using haplotype-based summary statistics. One advantage of this training strategy is that it enables an assessment of which data points are informative during training.

A comparison between the performance of FCNN and CNN to detect natural selection, specifically balancing selection, is presented by Isildak et al. (2021) in the software BaSe. Although both architectures exhibit high classification accuracy to distinguish between neutrality and selection, CNN outperformed FCNN to predict the type of balancing selection, a task that proved too challenging when relying solely on summary statistics as input. Authors used forward-in-time simulations and conditioned the target variants to a predefined range of final allele frequency. To counterbalance the increased computational time associated with this simulation scheme, a data augmentation to artificially enlarge the training data was adopted.

In recent years, the generation of sequencing data from ancient or historical samples, as well as from capture-recapture and evolve-and-resequence experiments, has allowed for a direct observation of how genetic diversity and allele frequencies change under natural or controlled conditions over time. To detect positive selection with time-series data, Whitehouse and Schrider (2022) proposed to stack either allele frequency or haplotype data over sampling times to be fed as input to one-dimensional CNNs. Their method was implemented in the software Timesweeper, and evaluated under various sampling conditions. Results show overall good accuracy levels for predicting selection, localizing the target variant, and distinguishing between selection from *de novo* mutation and from standing variation. Interestingly, using haplotype instead of allele frequency data yields a lower performance, possibly due to the difficulty in properly sorting the input data in a biologically meaningful way. Timesweeper was deployed to time-series pooled-sequencing data from *Drosophila simulans*, and it was able to replicate previously detected sweep signatures with better resolution.

CNNs have quickly become the main deep learning algorithm in population genetic studies thanks to their ability to automatically extract important features from raw genotype data, and their flexibility in accommodating different models to be tested. As a result, novel applications of such algorithms in population genetics are frequently proposed and introduced (Smith et al. 2022). In machine learning, natural language processing (NLP) represents a branch of algorithms that aims at “understanding” words in a text, meaning that they can, for instance, perform speech recognition, text generation, or sentiment analysis (i.e. associating an output label to each word or sentence). As DNA sequences are easily representable as a series of letters or motifs, in the next section, we will introduce NLP applications that are emerging in population genetics.

### Recurrent Neural Networks

Recurrent neural networks (RNNs) are algorithms derived from FCNNs but designed specifically for sequential data as they introduce a mechanism that influence current predictions based on previous outcomes (Minsky 1967; Rumelhart and McClelland 1987; Elman 1990). In fact, RNNs are comprised of connected nodes that form a cycle, with the output of some nodes feeding back to other (or same) nodes. Therefore, simple RNNs can be considered as for-loops iterating along the sequential data, where at each position the current input and the previous output are combined to form the next output (or hidden state). Multiple RNN layers can be stacked on top of each other to increase the capacity of the network and extract more features from the data. One of the limitations of RNNs is the limited capacity to learn long-range dependencies. Architectures such as Long Short-Term Memory (LSTM) and Gated-Recurrent Units (GRUs) networks circumvent this problem by adding the concept of cell state which is propagated along the sequence in the case of LSTMs, and GRUs enabling the filtering of passing information of long-range information through a Gating mechanism alone (Cho et al. 2014) whilst maintaining similar performance to LSTMs (Hochreiter and Schmidhuber 1997).

Recurrent layers have been used by Adrion et al. (2020) to estimate recombination maps for *D. melanogastor*. The proposed software ReLERNN provides a comprehensive modular workflow on how to generalize the method for different model species of interest, including instructions for phased, unphased and pooled-sequencing data. However, caution should be made when estimating recombination rates from genotype alignments using machine learning under certain conditions of low variability (Johnson and Wilke 2022). Hejase et al. (2021) proposed a method to detect natural selection by extracting features from estimated genealogical trees. They used counts of remaining lineages along a discrete log-transformation of the time dimension. The sequential nature of the trees along the sequence was used to set up an LSTM, which recognizes the lack of remaining lineages, that is zeros in the distant past or upper part of the feature matrix. This approach, implemented in the software SIA, gains the possibility to obtain an easily interpretable model at the cost of using an ancestral recombination graph (ARG)-inference method such as Relate (Speidel et al. 2019).

Inspired by the sequential nature of the Sequential Markov Chain (SMC) methodology, Khomutov et al. (2021) proposed an RNN method to estimate times to the most recent common ancestor from simulated data. Interestingly, this method achieved good results after coupling it with a CNN. Their approach is setup as a coalescent event classification strategy, thus creating a probability distribution of the TMRCA coalescent time at any given sequence position. Finally, neural net compression algorithms have been developed (Wang et al. 2018; Silva et al. 2020) making use of recurrent layers for the emphasis of long-range inter-dependencies and convolution layers. These approaches appear useful as the cost of sequencing dramatically decreases and becomes increasingly negligible compared with storage costs.

RNNs, in all their forms, have becoming increasingly popular in population genetics thanks to their ability to incorporate sequential data. Whilst training recurrent layers tend to be more challenging, coupling them with convolutional layers appear to be a suitable solution to overcome such issue whilst incorporating novel information. In the next section, we will explore how CNNs can be embedded in a more general family of machine learning algorithms called generative models.

### Generative Models

Generative models aim at capturing, and therefore approximating, the probability distribution between data and labels. By their nature, generative models are able to “generate” novel data points according to the captured probability distribution. Fitting a Gaussian mixture model and sampling from the distribution can be interpreted as a generative process, although it is insufficient to capture complex phenomena in high-dimensional spaces. In fact, even if sampling procedures can yield impressive results, that is for ARG inference (Mahmoudi et al. 2022), they often remain model-based, and are fundamentally limited by their run-time. For these reasons, deep generative models have become a subject of increased attention, especially for their capability of generating new samples even if the true underlying distribution is unknown. The following section focuses on three among the most popular non-model-based and high-parameter generative methods that have been explored in population genetics: autoencoders (Rumelhart and McClelland 1987), variational autoencoders (Kingma and Welling 2014), and generative adversarial networks (Goodfellow et al. 2014).

#### Autoencoders and Variational Autoencoders

Similar to Principal Component Analysis (PCA), autoencoders aim to solve a compression problem by step-wise reducing the input parameters into a smaller set of hidden parameters, analogues of the principal components. The number of hidden parameters, known as the latent space, is dependent on the network architecture. In a simple form, compression is achieved by an FCNN, called the encoder, with a decreasing number of learnable parameters in each layer. A second expanding network, called the decoder, rebuilds the original data from said latent space by minimizing a suitable loss function. An important part of the autoencoders is the regularization step, usually introduced as part of the loss function, which is necessary for learning a meaningful latent space by avoiding memorization.

Variational autoencoders (VAEs) differ from autoencoders as they introduce a generative operation by compressing the data into a latent space distribution, instead of a point representation. Furthermore, the latent space directly offers the possibility to probe the network for any kind of structure as input data, which the encoder has been forced to compress, by plotting the low-dimensional latent variables against each other. Thanks to the non-linearity of neural networks, VAEs outperform classic methods, that is PCA, for visual data representation (Battey et al. 2021).

VAEs have been implemented by Battey et al. (2021) in the software popvae. By applying it to genomic data sets, they recovered geographic similarities among human populations, and tested for robustness in the presence of genomic inversions in *Anopheles* mosquitoes. Additionally, low values of population genetic differentiation, as measured by $F_{ST}$ (Holsinger and Weir 2009), are more likely to be detected by VAEs. Lastly, whilst the generative property of VAEs has difficulties in detecting more complex relations, like long-range LD signatures, it can produce data with similar SFS patterns.

Other authors proposed a different VAE, named HaploNet (Meisner and Albrechtsen 2022) to infer population structure and ancestry proportions. HaploNet was shown to be able to infer parameters from very large genomic data sets, such as the UK Biobank and the 1000 Genomes Project. Likewise, others have proposed a multi-headed autoencoder, called Neural ADMIXTURE (Mantes et al. 2021), which was evaluated on the Simons Genome Diversity Project and the Human Genome Diversity Project, achieving similar results. Finally, López-Cortés et al. (2020) combined an autoencoder with common clustering methods, such as hierarchical clustering and K-Means. They sought to assign maize lines into subpopulations, and achieved marginally better results than by using a Bayesian clustering method.

#### Generative Adversarial Networks

Generative adversarial networks (GANs) provide a framework capable of estimating high-dimensional probability distributions by solving a min–max optimization problem between two opposing networks (Goodfellow et al. 2014). The aim of this architecture is thus to approximate the underlying data generation process (i.e. evolutionary process) of a study object of interest (i.e. genotype matrix). The model is capable then to *sample* new instances of the study object.

The first part of the architecture, called the generator network, only has access to the random distribution as a prior for constructing the target object, whereas the second network, called the discriminator has access to a real object (i.e. genotype matrix) and the generated object. The loss function from GANs illustrates the objectives of both networks: $L=E_{x}[\log\;(D(x))]+E_{z}[\log\;(1-D(G(z)))]$. The first part $E_{x}[\log\;(D(x))]$ representing the expected value of real samples x to be classified correctly by the discriminator (D(x)) and the second part $E_{z}[\log\;(1-D(G(z)))]$ stands for the expected value of generated data (G(z), z being the latent initialization) to be classified as fake by the discriminator (1−D(G(z))). Thus, the discriminator aims to maximize the loss function, whereas the generator tries to minimize it. The parameters of both networks are updated alternately. Optimization can be particularly challenging as neither network should be under-performing nor outperforming the other network too quickly. For instance, when both networks are not training *synchronously*, many values of the random initialization distribution can collapse into few target estimations, leading to decreased diversity of generated samples of the generator, a phenomenon known as the “Helvetica scenario” or “mode collapse” (Arjovsky and Bottou 2017). The discriminator would become trapped in a rejection space, and eventually end in a local minimum (Che et al. 2016). Another issue focuses around the fleeting convergence property during training, meaning the generator network becomes too good at misleading the discriminator, in which case the discriminator could only guess the correct class, resulting in poor gradients for both networks overtime.

In the first application of GANs in population genetics, Wang et al. (2021) integrated the coalescent simulator msprime (Baumdicker et al. 2021) with a parameter sampling algorithm (called simulated annealing) as the generator, with a CNN as discriminator. The objective was to infer optimal parameters of the simulations that generated realistic data sets. In this study, authors sought to estimate demographic parameters and recombination rate by evaluating both real and simulated data using summary statistics in a likelihood-free approach, similarly to ABC. In fact, authors compared their method, implemented in the software pg-gan, to an SFS-based ABC and achieved a similar performance. However, it is still unclear whether ABC or GANs yield a better performance in terms of the number and accuracy of parameters (here demographic changes), number of necessary simulations, and run-time for population genetic applications.

Beyond inferring parameters, the generative property of GANs has been explored in the form of other generative models such as Restricted-Bolthman-Machines (RBMs, Smolensky 1986; Teh and Hinton 2000). Yelmen et al. (2021) used RBMs to recreate a population structure data set as genotype matrices extrected from 1000 Genomes Project data set. The authors successfully demonstrated the ability of RBMs to reconstruct multi-modal distributions by reporting various distance measures (such as Wasserstein distance) and by visual inspection via dimensionality reduction. However, this initial attempt is not capable of recovering rare variant patterns, but advanced architectures designed to deal with mode collapse may solve this issue (Ghosh et al. 2017). Despite current limitations, GANs appear to be a promising deep learning framework to infer complex population genetic parameters in face of an uncertain or unknown demographic model (Booker et al. 2022).

## Available Resources

### Simulators

The application of deep learning methods has been empowered by decades of research into mathematical models of evolution and development of simulators built to recreate the hidden stochasticity of unseen evolutionary processes. In the context of deep learning, most of the applications in population genetics rely on training algorithms via synthetic data generated by such simulators. Broadly speaking, simulators can be categorized as forward-in-time and backward-in-time approaches. The latter category refers to coalescent simulators which, due to their rigorous underlying models, are extremely efficient as they only keep track of sampled genomes. Forward-in-time simulation tend to be more intuitive in their development, and are often used for complex selective processes which cannot be described by coalescent models. The following section is dedicated to name a few popular simulation tools, which can be used to generate data set to train neural networks.

SLiM (Messer 2013), provides a whole programming language Eidos (Haller 2016) designed to build forward simulation code for a vast range of evolutionary processes. Therefore, it has been used to train deep learning algorithms that aimed at inferring complex models. Interestingly, current developments on spatial simulators, such as slendr (Petr et al. 2022), leverage SLiM’s capabilities to generate synthetic genetic data variable in time and space. Likewise SLiM’s extensions to simulate bacterial populations (Cury et al. 2022) allow for studies of non-model organisms to generate synthetic data sets which could be used in a deep learning framework. Another forward-in-time simulator that has been used in deep learning is SFS_code (Hernandez and Uricchio 2015).

Among coalescent simulators, msprime (Baumdicker et al. 2021) is the preferred choice among practitioners due to its carefully designed code base, efficient tree sequence data structure (Kelleher et al. 2018), fast run-time, available choice of coalescent models (Adrion et al. 2020), easy programmatic access as well as active maintenance. It should be noted that tree sequences are not inherently limited to coalescent simulations, but have also been integrated into forward-in-time simulators such as SLiM (Haller et al. 2019), fwpyy (Thornton 2014) or sleepy (Korfmann, Abu Awad, et al. 2022). Lastly, ms (Hudson 2002), msms (Ewing and Hermisson 2010), fastsimcoal2 (Excoffier et al. 2021), and discoal (Kern and Schrider 2016) are coalescent tools that have been applied to train deep neutral networks for population genetic inferences.

### Software

Most of the studies herein mentioned provide their implementations, often as user-friendly software, of deep learning algorithms for population genetic analyses. In table 1, we summarize these implementations by the programming language and required (or preferred) simulator (if any) used, and by the input data required (table 1). We further categorize implementations based on their underlying type of neural network. Whilst general-purpose software for simulation-based inferences are available (Tejero-Cantero et al. 2020), here we focus only on implementations specific to population genetic analysis.

**Table 1:** List of Available Software and Implementations of Deep Learning Methods (not considering generative models) for Population Genetic Inferences

Reference	Language/Library	Simulator	Input
evoNet ${}^{a}$ (Sheehan and Song 2016)	Java	msms	Summary statistics
DeepGenomeScan ${}^{b}$ (Qin et al. 2022)	R/keras	Not trained by simulations	genotype, phenotype and sampling locations
Locater ${}^{c}$ (Battey et al. 2020)	python/keras	Not trained by simulations	Phenotype and sampling locations
ML_in_pop_gen ${}^{d}$ (Burger et al. 2022)	python/keras	msprime	SFS
ABC_DL ${}^{e}$ (Mondal et al. 2019)	Java/Encog and R/abc	fastSimcoal2	SFS
diploS/HIC ${}^{f}$ (Kern and Schrider 2018)	python/keras and scikit-learn	discoal	Summary statistics
partialS/HIC ${}^{g}$ (Xue et al. 2020)	python/keras and scikit-learn	discoal	Summary statistics
drosophila-sweeps ${}^{h}$ (Caldas et al. 2022)	python/pytorch	SLiM/msprime	Summary statistics
defiNETti ${}^{i}$ (Chan et al. 2018)	python/tensorflow	msprime	Genotype data
pop_gen_cnn ${}^{j}$ (Flagel et al. 2018)	python/keras	ms discoal	Genotype data
ImaGene ${}^{k}$ (Torada et al. 2019)	python/keras	msms	Haplotype data
dlpopsize ${}^{l}$ (Sanchez et al. 2021)	python/pytorch	msprime	Haplotype data
BaSe ${}^{m}$ (Isildak et al. 2021)	python/keras	SLiM	Haplotype data
genomatnn ${}^{n}$ (Gower et al. 2021)	python/tensorflow	SLiM	Genotype data
DeepSweep ${}^{o}$ (Deelder et al. 2021)	python/keras	SFS_code	Haplotype data
Timesweeper ${}^{p}$ (Whitehouse and Schrider 2022)	python/keras	SLiM	Haplotype or allele frequency time-series data
disperseNN ${}^{q}$ (Smith et al. 2022)	python/keras	SLiM or msprime	Genotype or tree sequence data and sampling locations
ReLERNN ${}^{r}$ (Adrion et al. 2020)	python/tensorflow	msprime	Genotype data
SIA ${}^{s}$ (Hejase et al. 2021)	python/keras	SLiM or discoal	Local trees
DNADNA t (Sanchez et al. 2022)	python/pytorch	msprime	Haplotype data

Note.—Software is gratefully supplied at their respective repositories: ${}^{a}$https://sourceforge.net/projects/evonet, ${}^{b}$https://xinghuq.github.io/DeepGenomeScan, ${}^{c}$https://github.com/kr-colab/locator, ${}^{d}$https://github.com/fbaumdicker/ML˙in˙pop˙gen, ${}^{e}$https://github.com/oscarlao/ABC˙DL, ${}^{f}$https://github.com/kr-colab/diploSHIC, ${}^{g}$https://github.com/xanderxue/partialSHIC, ${}^{h}$https://github.com/ianvcaldas/drosophila-sweeps, ${}^{i}$https://github.com/popgenmethods/defiNETti, ${}^{j}$https://github.com/flag0010/pop˙gen˙cnn, ${}^{k}$https://github.com/mfumagalli/ImaGene, ${}^{l}$https://gitlab.inria.fr/ml˙genetics/public/dlpopsize, ${}^{m}$https://github.com/ulasisik/balancing-selection, ${}^{n}$https://github.com/grahamgower/genomatnn, ${}^{o}$https://github.com/WDee/Deepsweep, ${}^{p}$https://github.com/SchriderLab/timeSeriesSweeps, ${}^{q}$https://github.com/kr-colab/disperseNN, ${}^{r}$https://github.com/kr-colab/ReLERNN, ${}^{s}$https://github.com/CshlSiepelLab/arg-selection, ${}^{t}$https://mlgenetics.gitlab.io/dnadna

From this collection, we note that recent implementations often rely on python packages such as keras and tensorflow which allow for easy building of layers, efficient optimization of networks, and intuitive monitoring of training performance. Implementations based on pytorch (another popular python package) allow for more flexibility in constructing complex architectures and investigating internal nodes. These python packages are supported by a strong and active community of developers and users, which ensures constant debugging and development.

We also note that forward-in-time simulators are becoming increasingly popular for training deep neural networks despite their significant computational cost, although the adoption of tree-sequence data and “simulation-on-the-fly” techniques can reduce such burden. Despite the plethora of implementations, each one appears to be suitable to perform specific tasks. At the moment of writing, only DNADNA (Sanchez et al. 2022) is the sole software providing a general framework to both generate simulations and build and training arbitrary networks.

## A Novel Application: Detecting Short-Term Balancing Selection from Temporal Data

We now wish to illustrate the feasibility and accessibility of deep learning algorithms to perform population genetics predictive tasks which are typically unachievable using classic approaches. To this aim, by using some of the architectures and techniques described above, we seek to develop a novel algorithm to detect signals of recent balancing selection from temporal genomic data.

Balancing selection is a process that generates and maintains genetic diversity within populations (Charlesworth 2006) whose signals are typically detected by investigating patterns of genetic diversity, allele frequency, and shared polymorphisms between species and populations (Key et al. 2014). Long-term balancing selection has been proved to be a major determinant of important phenotypes, including in humans (Soni et al. 2022). However, recent and fleeting balancing selection leaves cryptic genomic traces which are hard to detect and greatly confounded by neutral evolutionary processes (Sellis et al. 2011). Therefore, currently employed methods are either unsuitable or underpowered to detect short-term balancing selection (Fijarczyk and Babik 2015).

Information from temporal genetic variation, either from evolve-resequence or ancient DNA (aDNA) experiments, is particularly suitable to identify when and at to what extent natural selection acted (Dehasque et al. 2020). Previous attempts to use deep learning to infer balancing selection from contemporary genomes (Isildak et al. 2021) and positive selection from temporal data (Whitehouse and Schrider 2022) suggest that training an algorithm that uses the haplotype information from both contemporary and aDNA data has high potential to characterize signals of recent adaptation (and thus recent balancing selection).

To illustrate the ability of deep learning to detect signals of recent balancing selection, we simulated a scenario inspired by available data in human population genetics. We simulated 2,000 50 kbp loci under either neutrality or overdominance (i.e. heterozygote advantage, a form of balancing selection) at the center of the locus, conditioned to a demographic model of European populations (Jouganous et al. 2017). We performed forward-in-time simulations using SLiM (Haller and Messer 2019), similarly to a previous study (Isildak et al. 2021). We imposed selection on a de novo mutation starting 10k years ago, with selection coefficients of 0.25% and 0.5%. We sampled 40 present-day haplotypes, and 10 ancient haplotypes at four different time points (8k, 4k, 2k, 1k years ago, mirroring a plausible human aDNA data collection).

We trained a deep neural network to distinguish between neutrality and selection. Using pytorch, we built a network comprising two branches. One branch receives present-day haplotypes and performs a series of convolutional and pooling layers with permutation-invariant functions. The other branch processes stacked ancient haplotypes at different sampling points, and both branches performing residual convolutions. The two branches are merged with a dense fully layer that performs a ternary classification. We used 64 filters with 3x3 kernel size and 1x1 padding size after sorting haplotypes by frequency (Torada et al. 2019). We performed 10 separate training operations to obtain confidence intervals in accuracy values. We report results in the form of confusion matrices, a typical representation to summarize the predictive performance at testing. To further showcase the accessibility of deep learning, we made the full implementation and scripts are available at https://github.com/kevinkorfmann/temporal-balancing-selection.

**Fig. 3.**
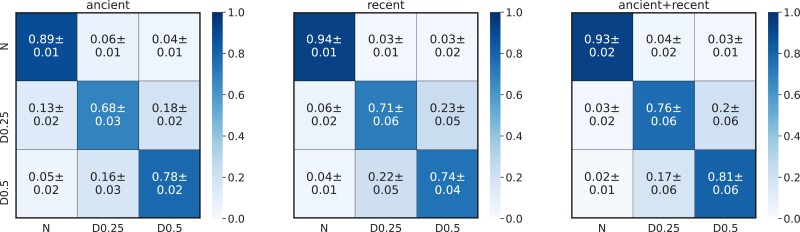
Confusion matrices to classify neutrality (N), weak (D0.25), or moderate (D0.5) overdominance with a deep learning algorithm using only ancient, present-day, or both types of samples. True and predicted classes are on the *x* axis and *y* axis, respectively.

Results show that, despite the small training set used, the network has high accuracy to infer recent balancing selection under this tested scenario (fig. 3). Notably, we observe a significant decrease in accuracy for distinguishing between weak and moderate selection when silencing the time-series branch, suggesting an important contribution of ancient samples in the prediction. In this illustrative example, we do not attempt to take into account the uncertainty given by degraded and low-coverage aDNA data and population structure across time points, among other confounding factors. Nevertheless, these results demonstrate that building and training novel deep learning algorithms is accessible and generates powerful predictions to address current questions in population genetics.

## Interpretable Machine Learning

As already mentioned in the Introduction, population genetics and evolution in general are aimed at uncovering the mechanisms responsible for the diversity of life in our planet. Thus, the black-box nature of deep learning methods represent an important obstacle for their application in these research fields. However, very recent advances in “interpretable machine learning” algorithms (Linardatos et al. 2021) are providing the tools needed to overcome this hurdle.

But what exactly do we mean by interpretability? There is no general consensus on what the word “interpretability” means (Doshi-Velez and Kim 2017; Fan et al. 2020) and discussions of this concept in the artificial intelligence literature tend to be rather abstract and sometimes highly technical. In the context of machine learning, a common definition is “the ability to explain or present in understandable terms to a human” (Doshi-Velez and Kim 2017). This abstract definition has been translated into a myriad of different operational definitions based on a wide range of criteria. In fact, several taxonomies for interpretability of neural networks have been proposed and the number of published articles on interpretability has been increasing exponentially since 2000 (Fan et al. 2020). Therefore, here we will restrict ourselves to distinguishing between global and local interpretability and explaining the relevance of these two concepts for population genomics studies. Also, we note that we will not consider very recent efforts aimed at designing inherently interpretable deep neural networks (e.g. Chen et al. 2020) and instead focus on post-hoc interpretation methods, that is algorithms that can be used to interpret an already trained network.

***Global interpretability*** aims at explaining the overall behaviour of a model (Ancona et al. 2019), which in turn can inform us about the system being studied. In principle, this goal can be achieved by analysing the hyperparameters (which control the learning process and the values taken by the parameters; for example learning rate, activation function, number of hidden layers, number of neurons per hidden layer) or parameters (weights and biases) of a deep neural network. However, the information provided by hyperparameters tend to be limited to model complexity, for example, in terms of the number of nodes and hidden layers retained after tuning and fitting or the type of activation function. On the other hand, the values taken by parameters (weights and biases) after fitting can provide more meaningful biological information; in particular, they help identify the features that contributed the most to the predictive power of the algorithm. For example, Sheehan and Song (2016) (see FCNN section above) use random permutation of each summary statistic (feature) and identify as most informative for the detection of population size changes those statistics that, when randomly permuted, lead to the sharpest decrease in accuracy. Another approach is based on feature importance (Olden and Jackson 2002), which was used by another study (Qin et al. 2022) to identify as outlier loci those that contributed the most to the power of an FCNN to predict an individual’s phenotype or geographic origin. Feature importance is based on the idea that the magnitude of connection weights between neurons connecting input and output nodes measure the extent to which each feature contributes to the network’s predictive power. The architecture used for these two examples was an FCNN. A different approach is necessary in the case of CNNs. For example, in the case of a CNN that classify images into different categories, a common approach is to use saliency maps, which measure the support that different groups of pixels in an image provides for a particular class (Mohamed et al. 2022). This is implemented by feeding the CNN an image of a particular class and using visualization techniques to generate heatmaps overlayed on the original image; the image elements that are being used by the CNN to identify the class are highlighted in red. A population genetics application of this approach is presented by Gower et al. (2021), who used a CNN algorithm to detect adaptive introgression.

***Local interpretability*** aims at understanding the reasons for a specific decision concerning a particular instance. Note that the ability of a particular feature to predict an attribute (e.g. phenotype) for a particular instance (data point), may depend on the values taken by the other features. This is particularly relevant in population genomics applications as the effect that a particular locus variant has on the phenotype of an individual may depend on the variants found at other loci (i.e. the genetic background; Chandler et al. 2013). A very promising technique to address this important issue is the Shapley value approach (Strumbelj and Kononenko 2010). Shapley values were first introduced in cooperative game theory (Shapley 1953) to calculate the contribution of individual players to the outcome of a game. In the context of deep learning, each feature represent a player, different combinations of features (feature subsets) represent a coalition, and the set comprising all features represents the “grand coalition of players”. The objective is to explain how values of a feature for a particular instance contribute to the difference between the prediction of a machine learning algorithm with the feature included and the expected prediction when the feature value is ignored (Strumbelj and Kononenko 2010). Thus, the Shapley value of a feature can be interpreted as the average marginal contribution of the feature to all possible feature subsets that can be formed without it (cf. Ancona et al. 2019). An important advantage of the approach is that it is the only explanation method that takes into account all the potential dependencies and interactions between feature values (cf. Strumbelj and Kononenko 2010). In principle, this requires the evaluation of all $2^{N}$ feature subsets (coalitions), were N is the number of features in the full set (grand coalition). Obviously, this is only possible when the number of features is small to moderate (some few dozens). Thus, several algorithms have been proposed for approximating Shapley values and a unified approach proposed by Lundberg and Lee (2017) has been implemented in both python (KernelShap and DeepShap) and R (shapr). However, they are limited to deep neural networks with moderate number of features. Nevertheless, very recent developments have led to new approaches, DASP (Ancona et al. 2019) and G-DeepShap (Chen et al. 2022), that may scale up to population genomics datasets. For the moment, there are no applications of Shapley values to population genomics studies; there is only an application in population genetics but in the context of random forests (Kittlein et al. 2022).

Much work remains to be done in order to incorporate the latest advances in interpretable machine learning to population genomics. Interpretability can lead to important breakthroughs by uncovering complex genomic signatures left by the non-linear interactions among many genetic and evolutionary processes. Although population genetics theory has already provided a deep understanding of the genomic signatures left by complex demographic history and selective processes, the “agnostic” nature of deep learning has the potential to uncover “hidden” genomic signatures that traditional model-based statistical methods are unable to detect. In doing so, they may generate new hypotheses for explaining observed genomic patterns that could then be tested.

## Dealing with Uncertainty

Whilst, as described so far, deep learning has led to novel applications in population genetics, the intrinsic challenges associated with uncertain DNA sequencing data, simulated training data sets, and an incomplete statistical framework are limiting factors to fully exploit the power of such technique.

As previously described, data given as input to deep learning algorithms in population genetics typically consist of alignments of genotypes, inferred haplotypes, or summary statistics. Genotype calling, phasing, and calculation of summary statistics are associated with statistical uncertainty (Nielsen et al. 2011), especially when performed from low-coverage sequencing (i.e. from museum specimen, ancient samples, or generally non-model species) (Lou et al. 2021). Sequencing data uncertainty could be tackled by providing estimates of summary statistics from genotype likelihoods as input. Additional approaches based on filtering masks to take into account data errors and missingness have been proposed in the literature (Adrion et al. 2020). Finally, generating sequencing data-like simulations (Escalona et al. 2016; Cury et al. 2022) for training could be a valuable solution to accommodate all nuances of the experimental data, at the expense of increasing computational resources needed. Other sequencing technologies may provide data of different nature [e.g. sample allele frequencies from pooled-sequencing experiments (Anand et al. 2016)], and therefore appropriate considerations should be made in terms of additional statistical uncertainty associated with such output. Approaches based on using trees or local ancestry tracts as input (Hamid et al. 2022) may be more prone to input data uncertainty.

One of the main concerns about current applications of deep learning in population genetics is the use of synthetic data for training neutral networks. For instance, the detection of signals of natural selection typically requires the knowledge of the underlying demography model to generate a null distribution under neutrality (Nielsen 2005). If the baseline demographic model is ill defined, inference of natural selection is expected to be biased (Johri et al. 2022). Whilst such issue is shared with other popular inferential frameworks, such as ABC (Bertorelle et al. 2010), the use of simulations in this context appears to be more problematic given the ‘black-box‘ nature of neural networks. Solutions to address the uncertainty of simulations explored in the literature include testing a network trained on misspecified models (e.g. Flagel et al. 2018; Torada et al. 2019; Adrion et al. 2020), and deploying it on known cases of selection and neutrality (Isildak et al. 2021) to quantify false positive and false negative rates. Although post-inference diagnostic analyses are required to ensure robustness of results, as per best-practice in machine learning (Lones 2021; Whalen et al. 2022), the ever-increasing curated list of demographic models (Adrion et al. 2020) will facilitate the use of synthetic data for training networks. Likewise, these resources will facilitate the establishment of gold-standard data sets to benchmark newly proposed architectures. Finally, efforts towards the adoption of transfer learning and domain adaptation techniques should further reduce any bias associated with uncertain training data sets.

Most applications described herein aim at classifying data into discrete labels or providing point-estimates of parameters of interests. Statistical uncertainty should be quantified by characterizing probability distributions of both the model uncertainty (epistemic or reduce-able part) and the inherent stochastic uncertainty of data generating process (aleatoric or irreduce-able uncertainty) (Hüllermeier and Waegeman 2021; Sanchez, Caramiaux, et al. 2022). Solutions to this problem include the prediction of mean and standard deviation (Chan et al. 2018) or confidence intervals alongside point estimates, and the quantification of any errors associated with the training phase (Smith et al. 2022). Thus, we encourage practitioners for the upcoming publications to consider modifying their models to account for uncertainty in a principled manner.

**From Regular Convolutions to Graph Convolutions**

Genotype matrices have been the starting point for doing any kind of population genetics analysis, either by calculating summary statistics (e.g. site frequency spectra), model-based probabilistic optimization algorithms (e.g. SMC), or Bayesian sampling techniques (e.g. ABC) and non-model-based function approximations (e.g. deep learning). Yet, recent trends emphasize a need to combine the power of deep learning approaches with a model-based constraint. A promising idea is to format the input data (genotype matrix) in order for model assumptions to be encoded directly in the data for subsequent training and inference. In the most general case, this model-based formatting can be considered as a representation of the ARG, for which few methods have been developed (Rasmussen et al. 2014; Kelleher et al. 2019; Speidel et al. 2019; Mahmoudi et al. 2022). Decoupling the ARG or genealogy construction and inference of evolutionary parameters of interest would create the opportunity to increase collaborations with mathematical modelers, by incorporating more complex coalescent models or biological processes like introgression, structured populations, or species-specific life-history traits. Additionally, it may no longer be necessary to try to interpret the inner workings of a CNN trained on (sparse) genotype matrices (which likely rebuilds parts of the ARG through complex aggregation of genotype density patterns). Any type of model-based properties could be questioned through modification of the ARG. An essential step has been developed by Korfmann et al., providing not only a new ARG-parameter inference method based on graph neural networks (GNN) but also an SMC method applied to a particular coalescent model, known for long-range LD interdependencies (Korfmann, Sellinger, et al. 2022). This approach offers the unique opportunity to test for mathematical model-based blind spots in an inherently Markovian constrained SMC method using GNNs.

### Conclusions

This review illustrates the great diversity of deep learning architectures that have been used in population genetics applications. Currently, the prevailing type of applications involve the training of algorithms with simulated data but there is an increasing number of studies that use a more standard approach where training is carried out using observed data. Thus, we can identify two strands of methods, one that is closely associated with likelihood-free, simulation-based approaches that consider explicit evolutionary models and another one that conforms to a purely data-driven, model-free approach. In both cases, however, deep learning is used as an inferential tool (as opposed to a predictive or pattern recognition approach). However, as the popularity of deep learning increases among population geneticists, we expect that further deep learning algorithms, including the latest diffusion models (Ramesh et al. 2022), will be adapted to solve predictive tasks. Intriguingly, novel applications may go beyond classic inferential tasks and include other aims, such as efficient data compression or generation of synthetic experimental data sets. Likewise, solutions for making neural networks a “transparent-box,” such as neural additive models (Novakovsky et al. 2022) and symbolic metamodeling (Alaa and van der Schaar 2019), will facilitate the adoption of deep learning among empiricists.

More research is needed in the domain of “interpretable” machine learning so as to gain an understanding of how deep learning algorithms make their decisions. This in turn would enable population geneticists to uncover novel genomic signatures associated with non-linear processes that current theory has not yet suggested including non-linear interactions among many genetic, ecological, and evolutionary processes. Importantly, further developments in local interpretability (see above) can help us to identify epistatic interactions and gain a better understanding of how genetic background influences the phenotypic effect of mutations.

One key aspect to make deep learning a popular framework in population genetics, is to ensure reproducible analyses and avoid repeating training of highly parameterized networks from scratch. In this context, recent efforts to provide users with documented workflows (Whitehouse and Schrider 2022) and pre-trained networks (Hamid et al. 2022) will both reduce carbon footprint (Grealey et al. 2022) and facilitate the application of deep learning to a wider range of data sets, allowing users to modify the network’s parameters according to the specific requirements of the biological system under examination.

Finally, we urge the community to make the field as inclusive as possible. Whilst open-source software release is common practice among machine learning practitioners, access to appropriate computing resources is still a limiting factor for many researchers. Initiatives to provide GPUs (i.e. graphics processing unit) and cloud computing credits to academics in need represent a valuable step towards making deep learning in population genetics accessible and inclusive to a wide range of scientists. Likewise, we encourage the establishment of training opportunities in machine learning for early-career population geneticists. Importantly, such events should happen either online or in hybrid format, with resources provided in multiple languages to ensure that text or verbal comprehension is not a barrier to learning. Consortia and local networks, properly funded by the wealthiest countries, appear to be a natural solution to fulfil this need. If all these conditions are met, deep learning will soon be established as part of the common toolkit among population geneticists globally.

## Data Availability

No new data were generated in support of this research. An implementation of the neural network illustrated in this review is available at https://github.com/kevinkorfmann/temporal-balancing-selection.
